# ZIF-8 Selective Dispersive Solid-Phase Extraction-LC-MS/MS Method for the Determination of Aconitine alkaloids in Rat Plasma: Application in Pharmacokinetic Studies

**DOI:** 10.1155/jamc/9937519

**Published:** 2025-02-26

**Authors:** Yang Yang, Liu Renyan, Xin Lingyi, Feng Baodong, Zhang Yu, Su Linqi, Ming Tingwen, Liu Jingjian, Chen Qinhua

**Affiliations:** ^1^Department of Pharmacy, Shenzhen Baoan Authentic TCM Therapy Hospital, Shenzhen 518101, China; ^2^Department of Pharmacy, Sinopharm Dongfeng General Hospital, Hubei University of Medicine, Shiyan 442008, Hubei, China; ^3^Department of Pharmacy, Shenzhen Bao'an Traditional Chinese Medicine Hospital, Shenzhen, Guangdong 518100, China

**Keywords:** aconitum alkaloid, d-SPE, Fuzi, pharmacokinetic study, ZIF-8

## Abstract

**Objective:** Aconitine alkaloids, as the principal bioactive constituents of Fuzi, pose a significant challenge to its clinical application due to their toxicity. This study aimed to establish a rapid, efficient, and stable method for quantifying monoester-type and diester-type alkaloids in raw Fuzi using zeolitic imidazolate framework-8 (ZIF-8). The method was subsequently applied to pharmacokinetic studies in rats, offering valuable insights into the safe clinical use of Fuzi.

**Methods:** Synthetic ZIF-8 was employed as the microextraction adsorbent, with optimization of extraction parameters such as ZIF-8 content, shaker speed, extraction time, and sodium ion concentration to maximize enrichment efficiency. A dispersive solid-phase extraction–liquid chromatography–tandem mass spectrometry (d-SPE–LC–MS/MS) method, based on ZIF-8, was developed and validated for method performance. The pharmacokinetics of five aconitine alkaloids in Fuzi were investigated, ensuring efficient extraction and analysis.

**Results:** Under the optimized conditions, the d-SPE method demonstrated robust enrichment of aconitine alkaloids. A strong linear relationship was established for aconitine, hypaconitine, mesaconitine, lappaconitine, and benzoylaconitine within the concentration range of 0.3125–1000 ng/mL, with correlation coefficients exceeding 0.99. The LC–MS/MS assay achieved a detection limit as low as 0.104 ng/mL. Additionally, the pharmacokinetic analysis revealed rapid absorption of the five alkaloids, with benzoylaconitine exhibiting a T_max_ of 0.25 h.

**Conclusion:** This study introduces a novel d-SPE–LC–MS/MS method based on ZIF-8 for the analysis of aconitine alkaloids in plasma, facilitating pharmacokinetic studies of Fuzi. These findings substantially contribute to a deeper understanding of the in vivo pharmacokinetics of aconitine alkaloids.

## 1. Introduction

The lateral roots of *Aconitum carmichaelii*, known as “Fuzi” in Chinese, exhibit a broad range of pharmacological activities, including antiarrhythmic [1], anti-inflammatory [2], analgesic [3], and antitumor effects [4, 5]. These Aconitum species have been used in traditional Chinese medicine (TCM) for over 2000 years and are officially recognized in the Chinese Pharmacopoeia. Over 600 effective TCM formulations containing Aconitum plants are widely used in clinical practice, such as the “Fuzi-Lizhong pill [6],” “Shenfu injection [7],” and “Jin-Gui-Shen-Qi wan [8].” Aconitine alkaloids present in Fuzi are both therapeutic and toxic components. Modern pharmacological studies have confirmed the effectiveness of aconitine alkaloids in treating cancer, pain, inflammation, and immune-related diseases [9]. However, aconitine, a highly toxic alkaloid [10], has a narrow therapeutic range [11], and its concentration in vitro does not accurately reflect its absorption in vivo. Consequently, accurate quantification of aconitine alkaloids (aconitine, hypaconitine, mesaconitine, lappaconitine, and benzoylaconitine) through pharmacokinetic analysis is essential for ensuring detoxification and the safe use of Fuzi.

**Figure 1:**
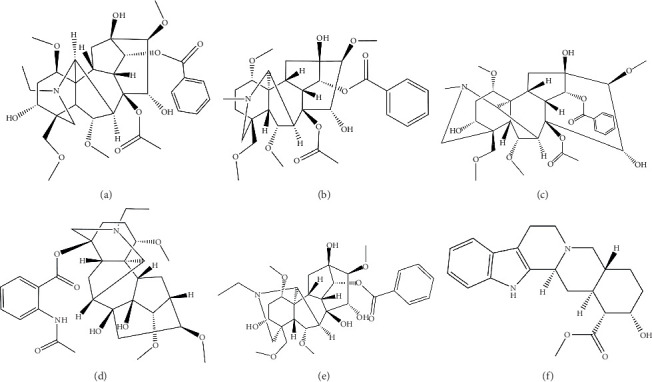
Molecular structures of (a) aconitine, (b) hypaconitine, (c) mesaconitine, (d) lappaconitine, (e) benzoylaconitine, and (f) the internal standard (IS) yohimbine.

The analysis of *aconitum alkaloids* (aconitine, hypaconitine, mesaconitine, lappaconitine, and benzoylaconitine; Figures 1(a), 1(b), 1(c), 1(d), and 1(e)) in Fuzi is essential for pharmacokinetic, pharmacodynamic, and toxicological studies. Despite extensive pharmacological research on *aconitine alkaloids* [12], systematic investigations into the pharmacokinetic characteristics and metabolic pathways of three of the five *aconitine alkaloids* remain limited. Some studies have reported rapid absorption of *aconitine alkaloids*, coupled with significantly low bioavailability [3]. Several analytical methods, primarily based on HPLC [13] and liquid chromatography–tandem mass spectrometry (LC-MS/MS), have been proposed for the quantification of these alkaloids [14]. For instance, Xu developed a novel HPLC-MS/MS method for the microextraction of aconitine, benzoylaconitine, and aconitine in water and plasma samples, achieving a detection limit of 0.49 ng/mL [15]. Zhang et al. investigated the biological distribution of three *aconitine alkaloids* in rat plasma, urine, feces, and various tissues following oral administration using LC-MS/MS, with a detection limit as low as 0.09 ng/mL [16]. To accurately monitor the levels of aconitine alkaloids in complex rat plasma samples, preanalytical steps involving proper sampling and sample preparation are essential for enhancing the efficacy of the analytical technique. These steps typically involve the removal of interfering matrix components and preconcentrating analytes to increase the sensitivity of the method. Dispersive solid-phase extraction (d-SPE) is particularly useful for purging impurities from biological samples while enriching aconitine alkaloids, making it a significant approach for the pharmacokinetic study of Fuzi and the precise determination of aconitine alkaloids in biological matrices.

d-SPE is a straightforward, rapid, and environmentally friendly sample preparation technique, rendering it highly suitable for the analysis of aconitine alkaloids in complex matrices of both plant and animal origins [17]. Zeolitic imidazolate frameworks (ZIFs), a subclass of metal-organic frameworks (MOFs) primarily composed of cobalt or zinc ions and imidazole ligands, are a class of porous materials with distinct advantages [18]. ZIFs inherit the beneficial characteristics of MOFs, including a rich pore structure, high specific surface area, structural diversity, and ease of modification. Additionally, ZIFs exhibit favorable biocompatibility and low toxicity [19]. These properties make ZIFs ideal candidates as carrier materials for d-SPE in the extraction of aconitine alkaloids. ZIF-8, for example, composed primarily of zinc ions and 2-methylimidazole, presents minimal toxicity to organisms, a large specific surface area, diverse structural features, adjustable pore sizes, and the ability to design and control surface properties, making it particularly effective in adsorbing alkaloid compounds [20, 21].

In this study, a novel ZIF-8-based d-SPE–LC method was developed for the quantification of five aconitine alkaloids in biological samples. The preparation of the monolithic sorbent was optimized for use in d-SPE, and extraction parameters were fine-tuned to achieve optimal extraction efficiency. Combined with HPLC, this analytical approach was established for the pretreatment and quantification of five aconitum alkaloid–related compounds in plasma samples. The method was subsequently applied to investigate the pharmacokinetics of Fuzi in vivo. To enhance the accuracy and precision of the method, yohimbine (Figure 1(f)) was used as an internal standard (IS). The method demonstrated high sensitivity, accuracy, and reproducibility, establishing its suitability for pharmacokinetic studies.

## 2. Materials and Methods

### 2.1. Materials

Processed Fuzi was provided by Kangmei Pharmaceutical Co., Ltd. (Chengdu, China) in May 2023. It was identified as the lateral root of *A. carmichaelii* Debx. The authentic sample (Fuzi KM 202305) is deposited at Shenzhen Bao'an Authentic TCM Therapy Hospital, Shenzhen, Guangdong, China. Aconitine (purity > 98.61%, lot: MUST-23030417), hypaconitine (purity > 98.59%, lot: MUST-23030319, confirmed by LC/MS/MS), mesaconitine (purity > 98.97%, lot: MUST-22120319, confirmed by LC/MS/MS), lappaconitine (purity > 97.69%, lot: MUST-23050119, confirmed by LC/MS/MS), and benzoylaconitine (purity > 98.09%, lot: MUST-22102110, confirmed by LC/MS/MS) were purchased from Chengdu Master Biotechnology (China). The IS, yohimbine (purity > 98.47%, lot: MUST-21072710, confirmed by LC/MS/MS), was also obtained from Chengdu Master Biotechnology. Zinc hexahydrate (Zn(NO_3_)_2_•6H_2_O), 2-methylimidazole (Hmim, C_4_H_6_N_2_), triethylamine (TEA), ammonium hydroxide (NH_4_OH), and acetic acid were of analytical grade. PBS (0.01 M, pH 7.2–7.4) was purchased from Beijing Solarbio Science & Technology Co. Ltd. Acetonitrile and methanol were obtained from Merck (Germany). Phosphoric acid of HPLC grade was sourced from Tianjin Kemiou Chemical Reagent Co., Ltd. Distilled water was provided by Guangzhou Watsons Food Co., Ltd., and hydrochloric acid was purchased from Dongguan City Dongjiang Chemical Reagent Co., Ltd. Sodium hydroxide was supplied by Tianjin Damao Chemical Reagent Factory.

### 2.2. Animals

Male Sprague–Dawley rats (250–300 g, 8–10 weeks old) were procured from Shenzhen Bao'an Authentic TCM Therapy Hospital (Guangzhou, China) and acclimatized in a controlled environment (temperature: 25°C ± 2°C; humidity: 50% ± 5%; 12-h dark–light cycle) for at least 3 days before experimentation (Animal License Number: SCXK [Guangdong] 2022-0002). Laboratory animal compound feed (Guangdong Feeding Certificate: 2019, No. 05,073) was provided. All animal experiments were performed in accordance with the Guide for the Care and Use of Laboratory Animals by the National Institutes of Health. Ethical approval for the animal studies was obtained from the Ethics Committee of Sinopharm Dongfeng General Hospital. The animal protocols used in this study were approved by the Institutional Animal Care and Use Committee of Sinopharm Dongfeng General Hospital.

### 2.3. Preparation of ZIF-8

Zn(NO_3_)_2_•6H_2_O (5.94 g) was dissolved in 30 mL of purified water. Then, 3.28 g of Hmim was added, followed by 11.12 mL of TEA. Afterward, 37.6 g (47 mL) of NH_4_OH was introduced, and the mixture was stirred until fully dissolved. The two solutions were combined and stirred vigorously for 10 min. The resulting mixture underwent suction filtration and was washed with purified water until a pH of 7 was achieved. The product was then dried overnight at 60°C to yield a white solid [22].

### 2.4. Instruments and Conditions

Instruments: The following instruments were used for the analysis: METTLER TOLEDO electronic analytical balance (1/100,000 tolerance); Sartorius water purification system (Sartorius [Shanghai] Trading Co., Ltd.); AB SCIEX 6500 + mass spectrometer (Shanghai AB Sciex Analytical Instruments Trading Co., Ltd.); Shimadzu LC-40D liquid chromatography (Shimadzu Co., Ltd., Japan); DA-type numerical control ultrasonic cleaner (Dongguan Kexiao Ltd.); DP-01 oil-free vacuum pump (Beijing Jiahang Bochuang Technology Co., Ltd.); R300 rotary evaporator (Shanghai Buchi Laboratory Equipment Trading Co., Ltd.); Rigaku SmartLab SE X-ray diffractometer (Japan); BET Micromeritics ASAP 2460 specific surface and porosity analyzer (USA); ZEISS GeminiSEM 300 scanning electron microscope (Germany); Hitachi HT7800 transmission electron microscope (Japan); and Thermo Fisher Scientific Nicolet iN10 Fourier Infrared Spectrometer (United Stated os Ameriva).

Chromatographic conditions were as follows: The analysis was conducted using an ACQUITY UPLC BEH HILIC C18 column (2.1 mm × 100 mm, 1.7 μm, Waters). The mobile phase consisted of 0.1% formic acid water (A) and methanol (B), with gradient elution as follows: 0–1 min: 90% A; 1–4 min: 90%–10% A; 4–8 min: 10% A; 8–9 min: 10%–90% A; and 9–10 min: 90% A. The flow rate was set to 0.4 mL/min, and the column temperature was maintained at 40°C. The injection volume was 3 μL. Mass spectrometry conditions were as follows: The analysis was performed using multiple reaction monitoring (MRM) in positive ion mode with an electrospray ionization (ESI) source. The ion source conditions were as follows: spray voltage: 5500 V; curtain gas (CUR): 20 psi; collision gas (CAD): low; ion source temperature: 550°C; ion source gas flow 1 (GS1): 55 psi; and ion source gas flow 2 (GS2): 55 psi. In this study, five components in crude Fuzi were quantified using ESI in both positive and negative ion modes. The intensity of the parent ions was higher in the positive ion mode, which was therefore chosen for the quantification. Optimal values for the declustering potential (DP) and collision energy (CE) were determined in MRM mode to achieve high-intensity peaks. The optimized mass spectrometry parameters for aconitine, hypaconitine, mesaconitine, lappaconitine, benzoylaconitine, and yohimbine (IS) were as follows: Ion pairs (m/*z*) were 646.300 ⟶ 586.300, 616.300 ⟶ 556.200, 632.400 ⟶ 572.200, 585.500 ⟶ 535.300, 604.500 ⟶ 554.400, and 355.200 ⟶ 212.300, with a CE of 46.000 V and a cluster removal voltage of 130.000 V.

### 2.5. Preparation of Samples

#### 2.5.1. Preparation of Fuzi Samples

The crude powder of Fuzi (50 g) was decocted in 500 mL of water for 1 h to obtain the crude extract, which was then concentrated to 100 mL and stored at 4°C.

#### 2.5.2. Standard and Quality Control Sample Preparation

Aconitine, hypaconitine, mesaconitine, lappaconitine, benzoylaconitine, and yohimbine were individually dissolved in 10 mL of methanol to prepare the stock solution. These compounds were subsequently diluted with methanol to achieve final concentrations of 10 and 5.0 μg/mL to prepare the working standard solutions, which were stored at 4°C before use. Under the optimized conditions, 10 μL of the working standard solution and IS (5.0 μg/mL), along with 15 mg of ZIF-8, was added to 200 μL of blank rat plasma and various blank tissue homogenates. The final concentrations of the calibration standard samples were 0.3125, 0.625, 1.5625, 3.125, 6.25, 12.5, 25, 50, 100, 500, and 1000 ng/mL. Plasma quality control (QC) samples were prepared at concentrations of 0.625, 6.25, 10, and 50 ng/mL. Standard calibration samples and HPLC–MS samples were stored at −20°C until analysis.

#### 2.5.3. Plasma Sample Preparation

**Figure 2:**
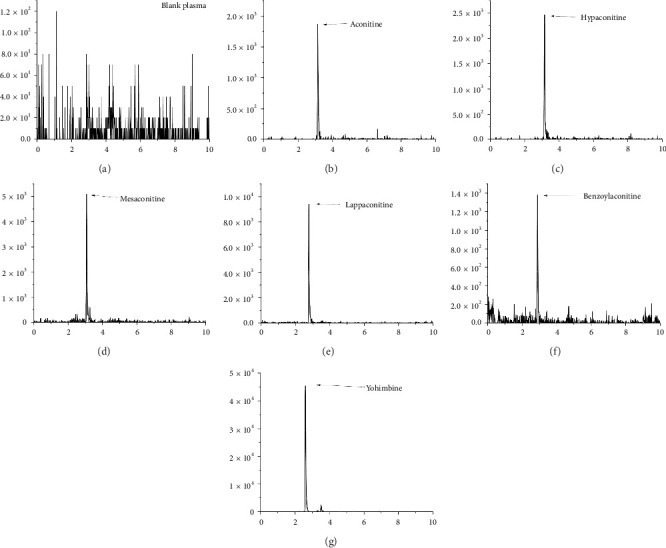
Representative HPLC-MS/MS chromatograms. (a) Blank plasma; (b) aconitine; (c) hypaconitine; (d) mesaconitine; (e) lappaconitine; (f) benzoylaconitine; (g) yohimbine.

All rats were maintained under standard conditions with ad libitum access to food and water. They were divided into two groups: Group 1 was used to prepare blank plasma samples, and Group 2 was designated for pharmacokinetic studies. Drug-free rat plasma was collected via suborbital venous hemorrhage. Pharmacokinetic studies involved continuous blood sampling from the suborbital venous plexus, as described previously. Each rat was administered a 10 mg/kg dose of Fuzi extract via gastric gavage. Blood samples (200 μL) were collected at 14 time points (0, 0.25, 0.50, 0.75, 1.0, 2.0, 3.0, 4.0, 6.0, 8.0, 10, 12, and 24 h postadministration) for pharmacokinetic analysis. The samples were collected into microtubes containing 5 μL of heparin, followed by centrifugation at 4000 rpm for 10 min. The resulting plasma (200 μL) was separated and immediately frozen at −20°C for further analysis.  Figure 2 illustrates the typical d-SPE–LC/MS profile of blank plasma and *aconitine alkaloids*, showing distinct peaks in rat plasma at 1 h postadministration without interference from endogenous peaks, thus maintaining a stable baseline for analyte and IS detection.

### 2.6. Extraction Process

**Figure 3:**
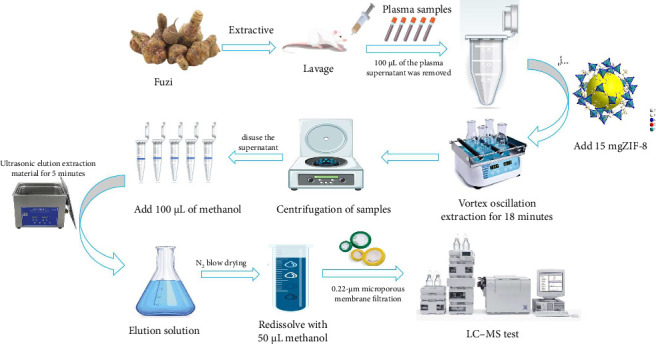
Flow chart depicting the extraction process of aconite alkaloids.

Before solid-phase extraction, ZIF-8 was activated by placing it in a vacuum-drying oven at 60°C for 24 h to remove surface-adsorbed water and other volatile substances. Rat plasma (100 μL) was combined with 350 μL of a 50% acetonitrile–water solution and 50 μL of yohimbine (IS). Following this, 15 mg of ZIF-8 was added to the mixture, which was then shaken for 18 min for extraction. After centrifugation, the adsorbent remained in the tube while the supernatant was discarded. Subsequently, 1000 μL of methanol was added to the tube, and ultrasonic elution of the nanocomposite was performed for 5 min. After centrifugation to collect the ZIF-8 nanocomposites, 900 μL of the eluent was evaporated using N_2_ flow at 40°C. The resulting residue was redissolved in 50 μL of methanol to obtain the final sample solution and analyzed by HPLC–MS/MS. The extraction process for *aconitine alkaloids* is depicted in Figure 3.

### 2.7. Pharmacokinetic Data Handling

The pharmacokinetic parameters of aconitine alkaloids in rats were calculated using Phoenix WinNonlin 8.3 software. The maximum plasma concentration (*C*_max_) and the time to reach Cmax (*T*_max_) were directly derived from the experimental data. The elimination rate constant (Ke) was determined by linear regression of the terminal semilogarithmic plot of plasma concentration versus time, and the elimination half-life (t1/2) was calculated as 0.693/Ke. All data are presented as mean ± standard deviation.

## 3. Results and Discussion

### 3.1. Characterization of Synthesized ZIF-8

**Figure 4:**
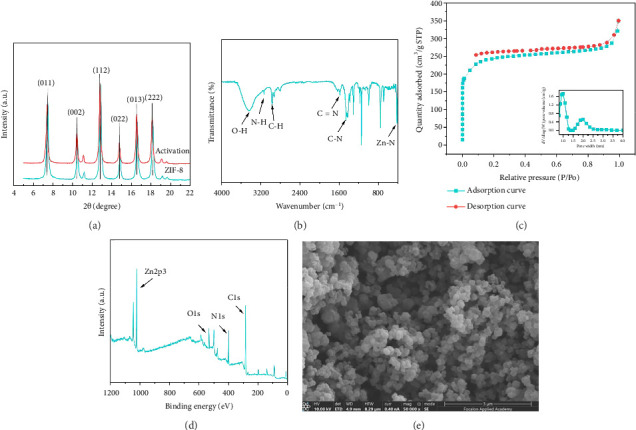
(a) PXRD characterization of materials; (b) infrared spectra; (c) N-adsorption–desorption isotherms; (d) X-ray photoelectron spectroscopy; (e) SEM images of ZIF-8.

The synthesized and activated ZIF-8 was analyzed using X-ray diffraction (XRD). As shown in Figure4(a), eight characteristic peaks at 2*θ* angles of 7.3°, 10.4°, 12.7°, 14.7°, 16.4°, 18.0°, 24.5°, and 26.7° confirm the successful synthesis of ZIF-8, which is consistent with previously reported data [23]. These diffraction peaks indicate that the synthesized sample possesses an ideal crystalline structure. Fourier transform infrared spectroscopy (FT-IR) analysis (Figure 4(b)) revealed absorption bands in the range of 3300–3000 cm^−1^, corresponding to the N–H stretching vibrations of 2-methylimidazole, confirming its successful coordination with zinc ions [24]. Additional characteristic absorption bands at 1620, 1440, and 421 cm^−1^ were attributed to C = N, C–N, and Zn–N stretching modes, respectively [25]. Notably, a broad and intense band at 3412 cm^−1^ indicated the presence of -OH functional groups [26]. These FT-IR results strongly support the successful coordination between 2-methylimidazole and zinc. The nitrogen adsorption–desorption isotherm (Figure 4(c)) demonstrated that the synthesized ZIF-8 exhibited a specific surface area of 907.57 m^2^/g, with a pore size distribution ranging from 0.8 to 2.5 nm, averaging 2.37 nm. X-ray photoelectron spectroscopy (XPS) analysis (Figure 4(d)) further confirmed the chemical composition and structural integrity of ZIF-8, with a binding energy peak for zinc observed at 1022.15 eV, consistent with literature reports [27]. Scanning electron microscopy (SEM) images (Figure 4(e)) revealed that ZIF-8 particles exhibited an irregular shape with varying sizes, forming spherical crystals with distinct edges.

### 3.2. Optimization of d-SPE Conditions

Given the instability and susceptibility to hydrolysis of aconitine alkaloids at high temperatures, shaker extraction was chosen as the preferred method. This technique allows for precise control over temperature and duration while being easy to operate. To optimize the d-SPE conditions, sample pH, ionic concentration, sample flow velocity, and sample volume were investigated. The sample volume was fixed at 1 mL, and the aconitine alkaloid concentration was set at 100 ng/mL.

**Figure 5:**
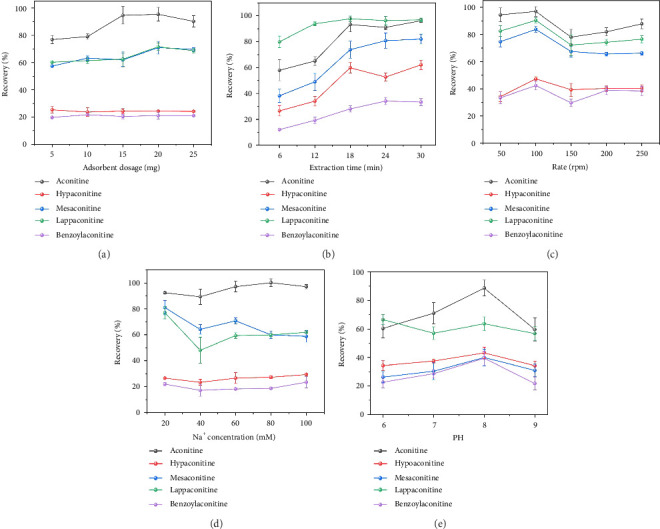
Optimizing extraction conditions. (a) ZIF-8 content; (b) extraction time; (c) rotation rate; (d) Na^+^ ion concentration; (e) pH.

This study systematically investigated the effects of ZIF-8 content, shaker speed, extraction time, Na+ concentration, and sample pH on the extraction efficiency of five aconitine alkaloids. The results are summarized in Figure 5. To optimize the adsorption capacity of ZIF-8 for these alkaloids, different amounts of ZIF-8 were compared (Figure 5(a)). Increasing the ZIF-8 content from 5 to 25 mg enhanced the adsorption of aconitine and mesaconitine, with saturation observed at 15 mg. However, hypaconitine, lappaconitine, and benzoylaconitine showed minimal changes in adsorption capacity across varying ZIF-8 contents. Therefore, 15 mg was identified as the optimal ZIF-8 content. The extraction time between 6 and 30 min was optimized (Figure 5(b)). For all five alkaloids, the best extraction efficiency was achieved at 18 min, after which the extraction appeared to saturate. Hence, 18 min was selected as the optimal extraction time. The influence of shaker speed on extraction efficiency was explored within a range of 50–250 rpm (Figure 5(c)). The results indicated that the adsorption of all five alkaloids gradually increased with higher speeds, reaching saturation around 100 rpm. Beyond this point, further increases in speed resulted in diminishing returns. Thus, 100 rpm was identified as the optimal shaker speed. The effect of Na+ ion concentration on extraction efficiency was also examined (Figure 5(d)). Increasing the Na+ concentration from 20 to 100 mmol/L had minimal impact on the adsorption of hypaconitine, lappaconitine, and benzoylaconitine. However, significant changes were observed for aconitine and mesaconitine, with the most effective extraction occurring at a 60 mmol/L Na+ concentration. pH values ranging from 6.0 to 9.0 significantly altered the distribution of binding sites on the ZIF-8 surface, consequently impacting the overall adsorption efficiency (Figure 5(e)). The extraction efficiency of aconitine alkaloids increased with pH, likely due to enhanced hydrogen bond formation. However, excessively basic conditions were found to be detrimental to ester bond formation in the aconitine alkaloid structure. Therefore, a pH value of 8.0 was chosen for further investigation. Considering these factors, the optimal extraction conditions for the five alkaloids were determined to be ZIF-8 content of 15 mg, shaker rotation speed of 100 rpm, extraction time of 18 min, Na+ concentration of 60 mmol/L, and pH of 8.0.

A suitable IS should exhibit structural or chemical similarity to the analyte, with comparable retention behavior and adequate separation from both the analyte and other peaks, while also mimicking the analyte during sample preparation. Yohimbine was selected as the IS for this assay due to its structural resemblance, retention characteristics, and binding affinity to aconitine alkaloids. Subsequently, liquid chromatographic separation conditions were optimized, with d-SPE parameters aligning with those previously determined.

### 3.3. Method Validation

Method validation was conducted following the guidelines set forth by the U.S. Food and Drug Administration for bioanalytical method validation (Bioanalytical Method Validation, 2001).

#### 3.3.1. Linearity and Limit of Detection

The calibration curve was constructed by calculating the ratio of peak areas of aconitine alkaloids to that of the IS, normalized to the theoretical concentration of the analyte. Data were analyzed using linear regression with 1/*x*^2^ weighting. Calibration standards were prepared at concentrations of 0.3125, 0.625, 3.125, 6.25, 12.5, 25, 50, 100, 500, and 1000 ng/mL. Linear calibration curves for aconitine alkaloids in rat plasma samples were established under optimal conditions (Table 1). The standard curve was fitted using the equation *y* = *ax* + *b*, where *y* represents the peak area ratio of aconitine alkaloids to the IS and *x* denotes the concentration of aconitine alkaloids (ng/mL), with *a* and *b* being constants. The weighted linear regression equation, derived from the peak heights of the analyte, resulted in a calculated limit of detection (LOD) of 0.1042 ng/mL, determined as 10 times the detector noise. The relative standard deviation (RSD) for trace detection of aconitine alkaloids in biological samples was found to be < 20%.

#### 3.3.2. Precision, Accuracy, and Recovery

The accuracy and precision of the method, evaluated by analyzing QC samples at three different concentrations, are summarized in Table 2. The intraday and interday RSD values ranged from 1.10% to 9.76% and 0.34%–9.78%, respectively. These results demonstrate that the method exhibits excellent reproducibility and precision.

#### 3.3.3. Analyte Stability

Stability tests were conducted to replicate real-world conditions that samples are likely to encounter. The results for plasma samples are summarized in Table 3. Storing QC samples at ambient temperature for up to 4 h before pretreatment had minimal effect on quantification. Samples stored at −20°C for up to 4 weeks and subjected to three freeze–thaw cycles maintained stability. The RSD for stability ranged from 0.33% to 9.91% in tissue samples stored at ambient temperature for up to 4 h. These results confirm that tissue samples can be stored at ambient temperature for up to 4 h without significant degradation.

#### 3.3.4. Pharmacokinetic Analysis

**Figure 6:**
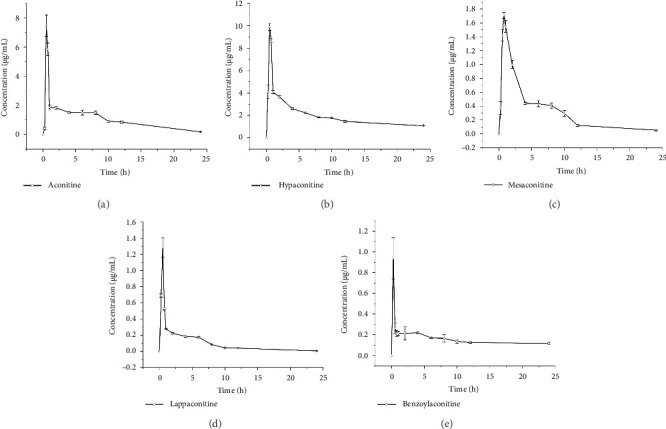
Mean plasma concentration–time curves. (a) Aconitine; (b) hypaconitine; (c) mesaconitine; (d) lappaconitine; (e) benzoylaconitine.

The plasma concentrations of five aconitine alkaloid species from Fuzi were quantified using the present method. The mean plasma concentration–time profiles are shown in Figure 6. Key pharmacokinetic parameters for these alkaloids were derived using a one-compartment model and are summarized in Table 4. The peak plasma concentrations of aconitine, hypaconitine, mesaconitine, lappaconitine, and benzoylaconitine were reached within 0.75 h, indicating rapid absorption into the systemic circulation. The half-lives (*t*_1/2h_) for these alkaloids were as follows: 5.7747 ± 0.3476, 21.2148 ± 0.5054, 5.3508 ± 0.5618, 3.8342 ± 0.7906, and 62.3852 ± 57.3582 h. These data suggest that aconitine, lappaconitine, and mesaconitine are eliminated more quickly than hypaconitine and benzoylaconitine. The maximum concentrations (*C*_max_) of aconitine, hypoaconitine, mesaconitine, lappaconitine, and benzoylaconitine were 7.4718 ± 0.7316, 9.9160 ± 0.3303, 1.7093 ± 0.0446, 1.2843 ± 0.1212, and 0.9391 ± 0.1994 ng/mL, respectively. This indicates a greater absorption of aconitine and hypaconitine compared with mesaconitine, lappaconitine, and benzoylaconitine. Notably, the *C*_max_ and AUC(0−*t*) values for mesaconitine, lappaconitine, and benzoylaconitine were significantly lower than those for aconitine and hypaconitine, suggesting that mesaconitine, lappaconitine, and benzoylaconitine face more challenges in absorption compared with aconitine and hypaconitine.

### 3.4. Adsorption Mechanism

**Figure 7:**
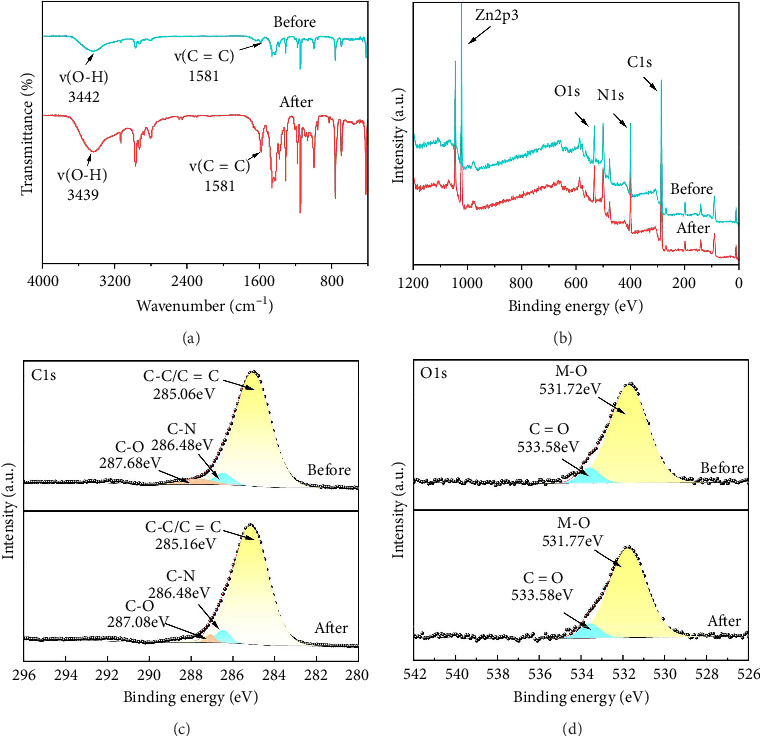
(a) Infrared spectra before and after adsorption; (b) XPS spectra before and after adsorption; (c) XPS fine peaks of C 1 s; (d) XPS fine peaks of O 1 s before and after adsorption.

The Zn–OH groups on the surface of MOFs are capable of forming hydrogen bonds with the O–H groups of aconitine alkaloids. Furthermore, the aromatic rings within the alkaloids can engage in *π*–π stacking interactions with the conjugated structures present in the MOFs. To clarify the adsorption mechanism of aconitine alkaloids on ZIF-8, FT-IR and XPS analyses were performed before and after adsorption.  Figure 7(a) presents the FT-IR spectra of the material pre- and postadsorption. After adsorption, the characteristic O–H group peak shifted from 3442 to 3439 cm^−1^, indicating the formation of hydrogen bonds. Additionally, the signal at 1581 cm^−1^ corresponding to C = C was intensified, further suggesting the presence of *π*–*π* conjugation interactions [28].  Figure 7(b) illustrates that the XPS spectra exhibited minimal changes before and after adsorption, indicating that the crystal structure of ZIF-8 remained intact. However, the Zn 2p_3/2_ peak shifted slightly from 1022.15 to 1022.20 eV postadsorption, which may reflect interactions between the surface material and the aconitine alkaloids [29]. In the C 1-s spectrum (Figure 7(c)), the peaks at 285.06 eV (C-C/C = C) and 287.68 eV (C-O) shifted to 285.06 eV and 287.08 eV, respectively, after adsorption. This shift is indicative of *π*–π conjugation interactions during the adsorption process [28]. In the O 1-s spectrum (Figure 7(d)), two distinct peaks were observed. After adsorption, the peak corresponding to metal oxide (M-O) shifted from 531.72 to 531.77 eV, suggesting that both oxidized metal states and carboxyl groups are involved in the interaction or adsorption of aconitine alkaloids onto the material's surface [30]. In conclusion, the adsorption mechanism of aconitine alkaloids on ZIF-8 is likely governed by a combination of hydrogen bonding and *π*–*π* interactions.

## 4. Conclusion

In the present study, the microextraction of aconitine alkaloids was performed using synthetic ZIF-8 as the adsorbent, and various parameters influencing the extraction efficiency were systematically evaluated. ZIF-8 exhibits good adsorption capacity for aconitum alkaloids through *π*–π interactions and hydrogen bonding. Under optimal conditions, the d-SPE method employing a shaker demonstrated excellent enrichment efficiency for aconitine alkaloids. A d-SPE–LC–MS/MS method was subsequently developed for the quantification of aconitine in plasma samples. The method's linear range spanned from 0.3125 to 1000 ng for aconitine, hypaconitine, mesaconitine, lappaconitine, and benzoylaconitine. The detection limit of the method by LC–MS/MS was as low as 0.104 ng/mL. This work marks the first application of ZIF-8 as an adsorbent in d-SPE, establishing a novel sample preparation technique combined with sensitive detection by HPLC, thereby contributing to the advancement of in vivo research on Fuzi. The analysis of aconitine alkaloids in rat plasma via d-SPE combined with HPLC highlights the rapid, sensitive, and straightforward nature of the d-SPE technique in sample pretreatment. Following d-SPE, the method exhibited a low detection limit, good linearity, and consistent results, fulfilling methodological requirements. These findings demonstrate the suitability of this approach for analyzing aconitine alkaloids in rat plasma and offer valuable insights for establishing blood drug concentration monitoring methods following the clinical use of Fuzi. The study also reveals that aconitine, lappaconitine, and mesaconitine are eliminated more rapidly than hypaconitine and benzoylaconitine. Aconitine, lappaconitine, and mesaconitine are diester-type diterpenoid alkaloids, whereas hypaconitine and benzoylaconitine are monoester or nonesterified forms. The faster clearance of the former group can be attributed to their heightened chemical reactivity and rapid hydrolysis through metabolic pathways. The molecular structure of diester-type alkaloids includes two ester groups, which confer greater chemical reactivity and increased susceptibility to hydrolysis. In contrast, monoester or nonesterified alkaloids exhibit greater structural stability and are less prone to hydrolytic degradation, leading to slower clearance. Upon entering the body, aconitine, lappaconitine, and mesaconitine undergo rapid hydrolysis in organs such as the gastrointestinal tract and liver, converting into less toxic monoester-type alkaloids or amine–alcohol derivatives that lack ester bonds. This transformation reduces toxicity and alters the physicochemical properties of the compounds, facilitating their excretion. Additionally, these metabolites may be more readily processed by hepatic enzymes, resulting in smaller molecules that are more efficiently eliminated. These mechanisms collectively ensure the swift hydrolysis, metabolism, and clearance of diester-type alkaloids, reducing their accumulation and potential toxicity. In summary, the rapid in vivo clearance of aconitine, lappaconitine, and mesaconitine is primarily due to their intrinsic chemical reactivity and efficient metabolic processing, which mitigate toxicity and promote their excretion. This understanding underscores the significance of considering the chemical structure and metabolic pathways of natural products when evaluating their safety and therapeutic potential in medicinal applications.

## Figures and Tables

**Table 1:** Regression lines of d-SPE.

Compound.	Equation	Linear range (ng/mL)	*R*
Aconitine	*y* = 28,003.78370*x* + 2855.94200	0.3125–1000	0.99273
Hypaconitine	*y* = 107,164*x* − 18,149.32693	0.3125–1000	0.99563
Mesaconitine	*y* = 50,098.2*x* + 60,642	0.3125–1000	0.99377
Lappaconitine	*y* = 174,849 + 7877.27312	0.3125–1000	0.99337
Benzoylaconitine	*y* = 3.7942*x* + 644.30290	0.3125–1000	0.99626

**Table 2:** Intraday and interday accuracy of Fuzi from biological samples.

Compd	c (ng/mL)	Precision RSD		Accuracy (Xz ± *s*, *n* = 5) %	
		Intrabatch	Interbatch	Intrabatch	Interbatch
Aconitine	0.625	1.10	3.18	97.52 ± 1.07	88.02 ± 2.80
	6.25	5.49	2.67	103.22 ± 5.67	100.65 ± 2.69
	10	9.63	7.75	89.46 ± 8.61	89.47 ± 6.93
	50	9.60	2.26	96.07 ± 7.25	85.55 ± 1.93

Hypaconitine	0.625	2.59	9.78	87.99 ± 2.64	86.10 ± 1.49
	6.25	4.17	6.39	91.35 ± 3.81	97.59 ± 6.24
	10	9.76	7.75	99.04 ± 7.91	95.95 ± 7.46
	50	4.01	9.40	96.12 ± 3.86	92.31 ± 8.68

Mesaconitine	0.625	8.05	8.64	94.13 ± 8.01	86.78 ± 2.36
	6.25	9.47	9.56	99.73 ± 8.95	96.42 ± 7.86
	10	3.37	7.28	99.45 ± 3.34	96.22 ± 7.60
	50	1.15	2.97	88.29 ± 1.01	93.95 ± 2.79

Lappaconitine	0.625	3.70	2.84	93.69 ± 3.47	88.46 ± 2.92
	3.125	7.01	2.11	86.49 ± 6.07	97.18 ± 2.10
	5	3.93	2.19	107.79 ± 2.48	100.89 ± 3.97
	25	1.38	0.34	100.50 ± 1.39	92.57 ± 0.32

Benzoylaconitine	0.625	2.64	6.04	98.99 ± 2.96	102.77 ± 6.20
	6.25	2.40	0.99	76.83 ± 1.84	76.11 ± 0.76
	10	1.89	0.73	99.95 ± 1.90	98.89 ± 0.72
	50	2.06	2.61	93.36 ± 1.92	83.37 ± 2.18

**Table 3:** Stabilization of aconitine alkaloids.

Compd	c (ng/mL)	Room temperature (8 h)		Automatic (24 h)		Freeze (−80°C for 30 d)		Freeze-thaw (three times)	
		RE %	RSD %	RE %	RSD %	RE %	RSD %	RE %	RSD %
Aconitine	6.25	−1.08	6.34	4.19	3.31	4.94	5.86	3.96	4.14
	50	−8.69	1.46	−10.01	2.07	−9.37	0.93	−8.46	1.02

Hypaconitine	6.25	−7.92	3.36	−8.00	5.72	−3.27	5.62	0.31	1.98
	50	−7.90	9.91	1.32	4.53	−2.50	5.26	3.47	2.41

Mesaconitine	6.25	0.97	3.41	−9.09	2.90	−7.23	8.08	−1.29	5.06
	50	−12.74	9.29	−9.89	3.73	−9.46	5.15	−7.50	4.98

Lappaconitine	3.125	−0.52	0.74	−0.82	0.33	−0.68	1.92	−2.04	0.51
	25	−6.67	1.04	−7.41	0.64	−9.65	1.01	−10.40	1.42

Benzoylaconitine	6.25	−7.89	0.82	−8.67	1.67	−7.70	0.67	−6.91	2.02
	50	−7.98	5.49	−9.65	0.44	−10.48	6.79	−10.57	5.88

**Table 4:** Estimated pharmacokinetic parameters for aconitine alkaloids in rat plasma (*n* = 6) after oral administration in rats.

Parameters	Units	Aconitine	Hypaconitine	Mesaconitine	Lappaconitine	Benzoylaconitine
t1/2	h	5.7747 ± 0.3476	21.2148 ± 0.5054	5.3508 ± 0.5618	3.8342 ± 0.7906	62.3852 ± 57.3582
*C*_max_	ng/L	7.4718 ± 0.7316	9.9160 ± 0.3303	1.7093 ± 0.0446	1.2843 ± 0.1212	0.9391 ± 0.1994
T_max_	h	0.5	0.5	0.75	0.5	0.25
AUC0-t	(ng/L)·h	25.3293 ± 0.5662	47.3490 ± 1.3325	7.6960 ± 0.4107	2.3983 ± 0.0276	3.7596 ± 0.0401
AUC0-∞	(ng/L)·h	26.9020 ± 0.6961	80.5104 ± 2.9061	8.1141 ± 0.4602	2.4210 ± 0.0223	14.0931 ± 9.3141
MRT0-t	h	7.1122 ± 0.1426	8.6272 ± 0.0519	5.6922 ± 0.1878	4.4699 ± 0.0814	9.9533 ± 0.3739
MRT0-∞	h	8.5864 ± 0.3488	27.5620 ± 0.5623	7.0326 ± 0.4791	4.7108 ± 0.2057	87.8556 ± 79.7064

## Data Availability

The data that support the findings of this study are available from the corresponding author upon reasonable request.
